# On the Use of Purgatives in the Treatment of Bilious Fevers, and Other Bilious Affections of the South and West

**Published:** 1853-04

**Authors:** 


### *On the Use of Purgatives in the Treatment of Bilious Fevers, and other Bilious Affections of the South and West.* By Sam'l G. Armor, M.D.

In withholding active purgation in the treatment of a class of diseases which prevails during the hot summer and the fall months, especially in the Southern and Western States, I am aware that I come in conflict with high authority; and I would not presume to question such authority, but for the conviction, strongly impressed upon my mind, that as a class of remedies, they are dangerous in the treatment of what are commonly called the Bilious, or Bilious Remittent Fevers of the South and West. It appears to be a common impression with many, that purgatives are the *only* remedies necessary in the treatment of this class of fevers.

It is not my purpose, at present, to inquire into the pathological relations of morbid hepatic secretions, further than as connected with diseased action of the gastro-intestinal mucous membrane. The general principles of pathology and practice, however, apply to all derangements of the hepatic functions.

Although lesions of secretions are generally classified by writers on pathology as *primary* elements of disease, yet a close examination of the subject must satisfy every reflecting mind that they are mere *symptoms*, or sustain secondary relations in the order of pathological manifestations. Before the lesion of secretion takes place, must there not be either a lesion of the blood, of the circulation, of structure or of innervation? A clear conception of this fact would, I think, throw light on a class of diseases associated with derangement of the hepatic function, and banish from our Nosology those numerous primary and idiopathic affections which are attributed to the liver.

It must be acknowledged, however, that as an excretory and depurating organ, the liver performs an important function in the animal economy; and the rationale of its increased action, and consequently increased stimulation, during the hot summer and fall months, must be apparent to every one who is familiar with the relation it sustains to the respiratory function. And the very importance of its office is a sufficient reason to induce us to investigate more closely its varied pathological conditions, that we may strike out, if possible, the first link in the chain of morbid action, and thus annul a train of secondary affections resulting from the forward action of a morbid secretion.

The peculiar tendency of Duodenitis to produce functional derangement of the liver has long been recognized by observers. The distinguished physiologist, Broussais, was the first, I believe, to call attention to this subject, and although he carried his views to great extremes, yet everlasting honor is due his memory for the clearness of his expositions of diseases of the gastro-intestinal mucous membrane. It is true, that so far as relates to the duodenal mucous membrane, different explanations have been given of the jaundice that so frequently follows. It has been supposed that a swollen condition of mucous membrane extending into the ductus communis choledochus, gives rise to mechanical obstruction to the flow of bile from the gall duct; and although in many instances this explanation may be the true one, yet the fact that we may have jaundice without closure of the common duct, is adverse to the universality of this explanation. We are led to infer, therefore, that the elements of the coloring matter of bile exist in the blood in health, and that other causes may impair or entirely suspend the secretory function of the liver; thus permitting the coloring matter to accumulate in the blood.

In cases of this kind, with the usual manifestations of an icterode appearance of the eyes and skin, and white or clay colored fæcal evacuations, we do not often have very marked tenderness over the region of the duodenum.

The question may arise, then—what is the morbid agency which gives rise to increased, suspended, or perverted action of the liver? The answer to this would show that the causes are various, although all agreeing, perhaps, in many essential particulars.

First, congestion from intropulsion of blood, whether from the cold stage of an intermittent fever, or from protracted cold to the surface, will give rise to it. The result of the congestion from any cause, whether active or passive, is the lowering of the vital properties of the gland, and a consequent suspension or perversion of secretion.

Again, perverted secretion may result from a primary diseased condition of the blood itself.

Or lastly, we may adopt the explanation of Bichat, "that between a secreting organ and the surface upon which its excreting duct opens, there is a sympathy by which a stimulus applied to the latter is communicated to the former." As applied to the liver, I should have enumerated this as first in the order of causes, because most important. The illustration of this law is very manifest. We have a familiar one in the effects of food, tobacco, or other stimulating substances taken into the mouth. A copious secretion from the salivary glands is the result. We have no explanation of this but that based on the influence of the sympathetic system of nerves over organic functions, and as applied to secretory organs, we have abundant evidence of this influence. Mental emotions also, such as anger, anxiety, fear and terror, very sensibly affect the secretion of glands. And so great is this perverted nervous influence, that it frequently affects, in a very marked degree, the quality as well as quantity of the secretion. Instances are on record, apparently well authenticated, of the secretions of the liver being rendered so acrid by violent emotions of anger, that at the moment of ejection it irritated the mouth, fauces and orifice of the anus. And the instance related in Carpenter's Physiology, of the violent combat between the soldier and the carpenter, whose wife was nursing a young infant, very forcibly illustrates the effect of passion in changing the secretion of the mammary gland. In our pathological reasonings we do not, perhaps, duly appreciate the influence which the great sympathetic system of nerves exercises over secreting structures.

These remarks are introduced for the purpose of showing that disordered hepatic secretion is a secondary condition, to be removed only by removing the cause. Hence, in a great practical point of view, the very important inquiry as to the nature of the cause.

But if from any cause, general or local, inflammatory or irritative, there is an interruption of the accustomed actions of a secreting organ, congestion of its vessels follows. As applied to the liver, diminished secretion of the bile gives rise to a congestive state of the vena portarum and its branches, and in some cases, to a similar state in those organs whose venous system is associated with that of the liver.

This organ, like all others, may be the seat of congestion, of inflammation, or of both. In speaking of congestion of the liver, I allude to a condition essentially different from inflammation of that organ. In acute inflammation, it is mainly the arterial action of that organ that is excited, and the congestion is arterial; whilst in venous congestion of the liver, consequent upon an interruption in its secretory action, the arterial system of the liver is necessarily but little, if at all affected; the congestive state of that organ being, in all probability, limited to the vena portarum and its branches. But from the peculiar vascular structure of the liver, while arterial determination must necessarily be followed by venous congestion, it can in no instance, as in other structures, contribute toward the relief of that condition.

If my premises then be correct, why do we administer cathartics for the relief of biliary derangement? I am aware that it is argued, theoretically, that the serous exhalation from the intestinal canal, caused by the action of a cathartic, unloads the vessels of the liver, and thereby restores its healthy circulation; and this argument might have weight, were it not for the counteracting influence of irritation, caused by the operation of the remedy; but this element of evil, I doubt not, more than overbalances all the benefit derived from the depletion. In many instances, the manifestations of biliary derangement are produced by irritation and phlegmasia of the mucous membrane; and it is very evident that this condition would be only exasperated by purgatives. An increased irritation is communicated to the parenchyma of the liver, and whatever be the intensity of the phenomena attributed to the bile, calmness, is generally re-established as soon as there is a cessation of the local phlegnasia. I regard this as an established fact in pathology of the highest importance.

In our ordinary bilious fevers, therefore, accompanied as they generally are with irritation of the stomach and bowels, I would abstain from the use of cathartics as calculated to aggravate the symptoms of biliary derangement and increase all the phenomena of the disease. I would not be understood, however, as entirely excluding alvine evacuants in the treatment of these fevers. Their operation is sometimes, doubtless, attended with benefit. The acrid secretion may be a greater source of irritation, forward upon the mucous membrane, and backward upon the gland secreting it, than would be the effect of a laxative to remove it; but it would be with this view, mainly, that I would administer them. The other fact, namely, that the tendency of cathartics is to increase the phlogosis of the mucous membrane, and that this condition is, through sympathy and direct continuity of structure, communicated to the liver, should ever be borne in mind.

If their effect be to indirectly at least stimulate the liver, our deduction may be regarded as illogical. The question may be asked, is not this the great object to be effected? Grant that it is, to say the least of it, a desirable object, and still it by no means follows that the enteric and hepatic excitement will be promotive of biliary secretion. Is not indeed the converse of it true? Yet there may be a possibility that the secretory action of the liver is suspended from the want of the normal sensibility of the duodenal mucous surface.

The chyle, which is the natural stimulus of this surface, may, as a consequence, fail to communicate its stimulatory impression to the liver, and a sort of torpor or paralysis may be the result. This condition is generally manifested by the clay-colored or white discharges from the bowels, unaccompanied by hyperæmia and tenderness. If we are able to diagnose this condition, then purgatives, especially the mercurial ones, may be admissible; although even in this case, broken doses of calomel, short of purgation, would be better practice.

Is it true, that in the class of cases under consideration these are the manifestations? Is not indeed the very opposite condition generally present, such as local tenderness, irritability of the stomach, and dark discharges, indicating morbid sensibility and hyperæmia of the mucous membrane to the point of effusion of the morbid and fluid elements of the blood?

Shall we, then, in this condition, administer cathartics? Many reasons forbid; I will be content with enumerating a few:

1st. As a general and valuable therapeutical principle we should never resort to medicinal agents when nature is doing her proper work.

2d. Cathartics will, in all probability, increase the very difficulty which nature is endeavoring to overcome, by adding irritation and determination to the congestion already existing.

3d. Protracted congestion of the liver, by damming back the venous circulation of the abdomen, may give rise to formidable disease of the intestinal mucous membrane.

And lastly, there is no indication, as a general proposition, for their use, as evinced by the color and character of the discharges characterizing hyperæmia and effusion, positively contra-indicating their use.

I might add, that experience abundantly demonstrates not only the inutility, but the positive injury following the use of active and repeated purgation in the treatment of the miasmatic fevers of the Mississippi Valley. I doubt not but that hundreds have fallen victims to erroneous views on this subject, propagated by Hamilton in his work on Purgatives.

I have alluded more especially to the use of cathartics in the treatment of ordinary bilious fevers, as they are generally termed, and have called attention to but one pathological feature of the disease. In so doing I would not be understood as referring all the phenomena of bilious fever to derangement of the biliary organs; nor to enteritis or gastro-enteritis as the cause; notwithstanding this is undoubtedly a frequent and formidable super-addition to the general fever.

The effect of cathartics is also bad on the gastric mucous membrane, and consequently on the functions of the stomach; and it is only necessary to reflect on the importance of the perfect action of the digestive apparatus to a maintenance of a healthy condition of the entire system, to be convinced of the multiplied variety of secondary disturbances which may result from derangement of the primary action of the series of animal functions. It is indeed the "golden bowl at the fountain," the "wheel at the cistern," and if its functions be perverted, disturbance is, of a physical necessity, propagated remotely through the system. Strike upon the first link of the chain of sympathies, and vibration runs through its whole extension. Hence the varied course which the derangement of function may pursue, and hence the difference of character which disease may ultimately assume. If this thought were more rigorously pursued in all investigations at the bedside, the result would doubtless be a more rational and simple practice.

Medicine has too often and long been engaged, and too often worsted, in the contest with affections of an idiopathic and independent character, which werethe secondary, or perhaps more remote result of pathological derangement; and in no instance, perhaps, have we a more striking illustration of this than in diseases of the gastro-intestinal mucous membrane.—*Ohio Med. and Surg. Journal, from Western Med. Chir. Journal.*

